# Analysis of risk characteristics for metachronous metastasis in different period of nasopharyngeal carcinoma

**DOI:** 10.1186/s12885-023-10641-8

**Published:** 2023-02-17

**Authors:** Zhaodong Fei, Huiling Hong, Ting Xu, Yiying Xu, Jiawei Chen, Xiufang Qiu, Jianming Ding, Ye Feng, Chaoxiong Huang, Li Li, Mengying Li, Chuanben Chen

**Affiliations:** Department of Radiation Oncology, Clinical Oncology School of Fujian Medical University, Fujian Cancer Hospital, Fuma Road, Fuzhou, 350014 Fujian People’s Republic of China

**Keywords:** Nasopharyngeal carcinoma, Distant metastasis, Interactive risk attributable program, Attributable risks, Metachronous metastatic nasopharyngeal carcinoma

## Abstract

**Objective:**

To identify the main risk factors for metachronous metastatic nasopharyngeal carcinoma (NPC) in different periods after radiotherapy and estimate the weight of various factors in the early or late metachronous metastasis (EMM/LMM) groups.

**Methods:**

This retrospective registry consists of 4434 patients with newly diagnosed NPC. Cox regression analysis was used to assess the independent significance of various risk factors. The Interactive Risk Attributable Program (IRAP) was used to calculate the attributable risks (ARs) for metastatic patients during different periods.

**Results:**

Among 514 metastatic patients, 346 (67.32%) patients diagnosed with metastasis within 2 years after treatment were classified into the EMM group, while other 168 patients were classified into the LMM group. The ARs of T-stage, N-stage, pre-Epstein-Barr virus (EBV) DNA, post-EBV DNA, age, sex, pre-neutrophil-to-lymphocyte ratio, pre-platelet-to-lymphocyte ratio, pre-hemoglobin (HB), and post-HB were 20.19, 67.25, 2.81, 14.28, 18.50, - 11.17%, 14.54, 9.60, 3.74% and - 9.79%, respectively, in the EMM group. In the LMM group, the corresponding ARs were 3.68, 49.11, - 18.04%, 2.19, 6.11, 0.36, 4.62, 19.77, 9.57 and 7.76%, respectively. After multivariable adjustment, the total AR for tumor-related factors was 78.19%, and that for patient-related factors was 26.07% in the EMM group. In the LMM group, the total AR of tumor-related factors was 43.85%, while the weights of patient-related factors was 39.97%. In addition, except for these identified tumor- and patient-related factors, other unevaluated factors played a more important role in patients with late metastasis, with the weight increasing by 15.77%, from 17.76% in the EMM group to 33.53% in the LMM group.

**Conclusion:**

Most metachronous metastatic NPC cases occurred in the first 2 years after treatment. Early metastasis was mainly affected by tumor-related factors, which accounted for a declining percentage in the LMM group.

**Supplementary Information:**

The online version contains supplementary material available at 10.1186/s12885-023-10641-8.

## Introduction

Nasopharyngeal carcinoma (NPC) is a malignant tumor that originates from the mucosal epithelium of the nasopharynx, is highly prevalent in Southeast Asian countries, and has obvious ethnic and regional distribution characteristics [1]. In 2020, approximately 133,000 new cases of NPC were diagnosed worldwide [2]. In the era of intensity-modulated radiotherapy (IMRT), locoregional control has improved considerably for NPC, and the primary reason for treatment failure was attributed to distant metastasis [3].

Distant metastases can be divided into synchronous and metachronous [4]. Approximately 10% of patients with NPC present with distant metastases at the first diagnosis, termed synchronous metastatic NPC. In addition, 10–20% of patients eventually progress to metastasis after treatment, which is termed metachronous metastatic NPC [5]. The time to metachronous metastasis differed significantly among the patients. However, there is no consensus on the appropriate time point to divide patients into those with early and late metachronous metastases.

Many researchers have investigated the clinical features and risk factors of metastasis in patients with NPC. Previous studies have demonstrated that several risk factors, such as the TNM staging system, age, plasma Epstein-Barr virus (EBV)-DNA copy numbers, hemoglobin (HB), neutrophil-to-lymphocyte ratio (NLR), and platelet-to-lymphocyte ratio (PLR), may be related to metastatic NPC [6–8]. However, few studies have comprehensively examined their contribution to metastatic burden. In addition, no study has investigated the difference and weight of risk factors for metastasis in different periods.

In this study, we attempted to determine an appropriate time point to divide patients into early and late metastasis groups. We then estimated the population attributable risks (PARs) for various risk factors identified in our large real-world retrospective study to further explore the differences and weights of risk factors for metastasis in different periods.

## Materials and methods

### Patient

This retrospective registry consists of 4434 patients with newly diagnosed NPC in our center between January 2012 and December 2018 (Supplementary Fig. 1). The inclusion criteria were as follows: (1) biopsy-proven primary NPC; (2) radical radiotherapy; (3) Karnofsky performance score = 80; and (4) complete medical records and clinical information, including adequate clinical examination and laboratory data. The exclusion criteria were as follows: (1) distant metastasis at first diagnosis, (2) second primary carcinoma, (3) severe medical complications, and (4) disrupted treatment. All patients were restaged according to the 8th edition of the American Joint Committee on Cancer (AJCC) Staging System. We investigated the clinical features and potential risk factors in these patients. The study was approved by the Ethics Committee of Fujian Cancer Hospital (no. YKT2020–011-01).

### Clinical feature

Based on previous studies and existing research results, we incorporated relevant factors in this study, including T-stage, N-stage, age, sex, pre (before treatment)-NLR, pre-HB, pre-PLR, pre-EBV DNA (plasma EBV DNA copy numbers), post (after chemoradiotherapy)-HB, and post-EBV DNA (plasma EBV DNA copy numbers). Pre- and post-EBV DNA levels were divided into two groups: 0 copy/mL and > 0 copy/mL. Subsequently, other continuous variables, including age, pre-NLR, pre-HB, pre-PLR, and post-HB, were transformed into categorical variables using medians. The median values were as follows: age, 50 years; pre-NLR, 2.045; pre-HB, 143 g/L; pre-PLR, 125; and, pre-HB, 122 g/L. The selected clinical factors were divided into two categories. Tumor-related factors included T-stage, N-stage, pre-EBV, and post-EBV, while patient-related factors included age, sex, pre-NLR, pre-HB, pre-PLR, and post-HB.

### Treatment

All patients were treated with IMRT with or without chemotherapy. The radiotherapy dose and target volume delineation were performed according to the institutional treatment protocol [9]. In brief, the total dose of planning target volume obtained for the gross tumor volume in the primary tumor or in the involved lymph nodes were 69.7–70.0 Gy in 33–35 fractions.

Stage I patients were treated with radiotherapy alone and stage II patients received 2 cycles of concurrent chemoradiotherapy (CCRT), while stage III-IV patients received 2–3 cycles of induction chemotherapy (IC) followed by 2 cycles of CCRT. In addition, adjuvant chemotherapy (AC) was given at the discretion of the radiation oncologists, not as the standard treatment.

### Follow-up

After completing the standard treatment, symptom inquiry, physical examination, routine blood tests, EBV DNA copy number in peripheral blood, fiberoptic endoscopy, nasopharynx and neck MRI, chest CT, and abdominal ultrasound were performed every 3 months for the first 2–3 years, every 6 months up to the fifth year, and annually thereafter. The time to metastasis was selected as the primary endpoint, which was defined as the duration from the date of NPC diagnosis to the date of the first metastasis.

### Statistical analysis

Statistical analysis was performed using the R software (version 4.1.3) and SPSS version 27.0 (IBM Corp., Armonk, NY, USA). The life-table method was used to calculate the annual distant metastasis rate. Baseline characteristics were compared using the chi-squared test for categorical variables. Cox proportional hazards regression analysis was used to assess the independent significance of the different metastatic factors. Odds ratios and 95% confidence intervals were calculated for individual risk factors and the combination of tumor- and patient-related risk factors based on an unconditional logistic regression model. The Interactive Risk Attributable Program (IRAP) (version 2.2) was used to calculate the attribution risk (AR) for patients in early or late metastasis groups [10, 11]. All tests were two-tailed, and statistical significance was set at *p* < 0.05.

## Results

### Patient characteristics and metastasis distribution

**Fig. 1:**
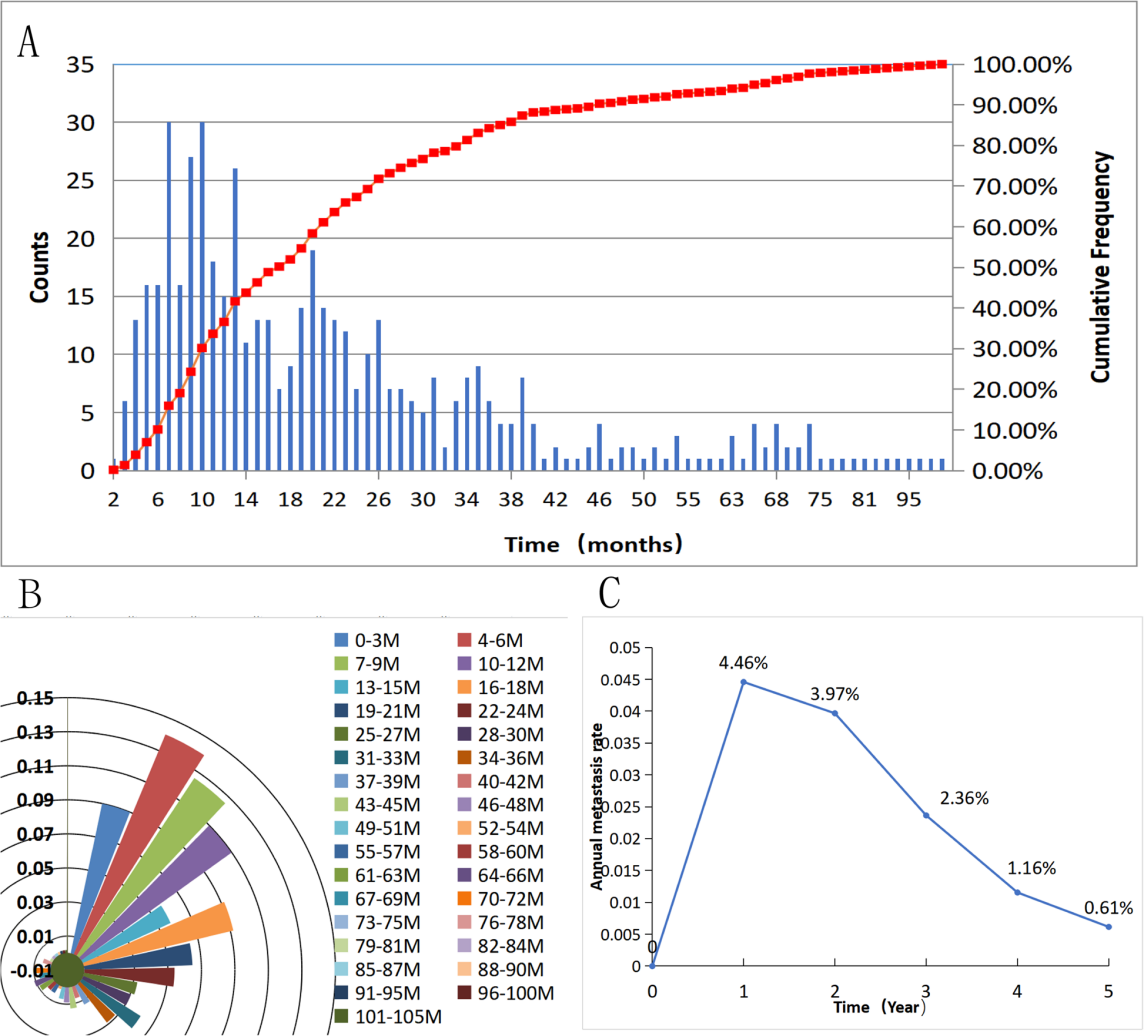
**A** Cumulative frequency distribution histogram for metachronous metastatic NPC. The left Y-axis stand for the counts of patients with metastatic NPC at different times. The right Y-axis stand for the cumulative frequency of distant metastases over time. The curve shows the change in the cumulative frequency of distant transitions over time. **B** Monthly distribution map of distant metastasis in NPC. This graph shows the counts of patients with metastatic NPC every 3 months as a proportion of the total number of patients with metastatic disease. **C** Annual distant metastasis rate in NPC

The clinical characteristics of all eligible patients are summarized in Table 1. Among the 4434 enrolled patients, 514 experienced metastasis after a median follow-up of 55 months (range, 1–119 months). Figure 1A shows that 67.32% (346/514) of patients experienced their first metachronous metastasis within 2 years after treatment. The proportions of distant metastasis in the first, second, third, fourth, and fifth years were 36.58% (188/514), 30.74% (158/514), 16.93% (87/514), 6.61% (34/514), and 2.53% (13/514), respectively (Fig. 1B). The distant metastasis risk curve for the entire cohort reached a peak in the first year, with an annual distant metastasis rate of 4.46%; then, the risk gradually decreased in the second year, with an annual rate of 3.97%. Finally, the risk of metastasis dropped dramatically at 3–5 years (Fig. 1C).

**Table 1:** Characteristic baseline table

Characteristic	*N* = 4434	Number of patients (%)
Sex	male	3215 (72.5)
	female	1219 (27.5)
Age (year)	≤50	2666 (60.1)
	>50	1768 (39.9)
T-stage	1	876 (19.8)
	2	902 (20.3)
	3	1506 (34)
	4	1150 (25.9)
N-stage	0	410 (9.2)
	1	1467 (33.1)
	2	1769 (39.9)
	3	788 (17.8)
Pre-NLR	<2.045	2198 (50)
	≥2.045	2201 (50)
Pre-HB (g/L)	≤143	2137 (48.6)
	>143	2263 (51.4)
Pre-PLR	<125	2215 (50.4)
	≥125	2184 (40.6)
Pre-EBV DNA (copy/ml)	0	3166 (74)
	>0	1115 (26)
Post-HB (g/L)	<122	2085 (47.2)
	≥122	2330 (52.8)
Post-EBV DNA (copy/ml)	0	3561 (88.5)
	>0	465 (11.5)

*Abbreviations*: *Pre-NLR* neutrophil to lymphocyte ratio before treatment, *Pre-HB* hemoglobin before treatment, *Pre-PLR* platelet to lymphocyte ratio before treatment, *Pre-EBV DNA* Epstein-Barr virus DNA before treatment, *Post-HB* hemoglobin after chemoradiotherapy, *Post-EBV DNA* Epstein-Barr virus DNA after chemoradiotherapy

Based on the metastasis distribution, all patients with metastatic disease were divided into two groups. A total of 346 patients with first metachronous metastasis within 2 years of treatment were classified into the early metachronous metastasis (EMM) group. A total of 168 patients with first metachronous metastasis more than 2 years after treatment were classified into the late metachronous metastasis (LMM) group. The remaining 3920 patients without metastasis were defined as the non-metachronous metastasis (NMM) group.

### Identify the risk factors for metastasis

Table 2 shows the difference in the risk factors among the three groups, including sex (*p* = 0.009, NMM VS. EMM; *p* = 0.079, NMM VS. LMM), age (*p* = 0.011, NMM VS. EMM), T-stage (*p* = 0.007, NMM VS. EMM), N-stage (*p* < 0.001, NMM VS. EMM; *p* < 0.001, NMM VS. LMM), pre-NLR (*p* = 0.001, NMM VS. EMM), pre-HB (*p* = 0.059, NMM VS. EMM; *p* = 0.012, NMM VS. LMM), pre-PLR (*p* = 0.067, NMM VS. EMM), pre-EBV DNA (*p* = 0.002, NMM VS. EMM), and post-EBV DNA (*p* < 0.001, NMM VS. EMM).

**Table 2:** Baseline characteristics and chi-square test for NMM, EMM and LMM group

Characteristic	NMM group (*n* = 3920)		EMM group (*n* = 346)		NMM VS. EMM	LMM group (*n* = 168)		NMM VS. LMM
Sex					0.009			0.079
Female	1107	28.20%	75	21.70%		37	22.00%	
Male	2813	71.80%	271	78.30%		131	78.00%	
Age (year)					0.011			0.532
≤50	2332	59.50%	230	66.50%		104	61.90%	
>50	1588	40.50%	116	33.50%		64	38.10%	
T-stage					0.007			0.683
1	789	20.10%	54	15.60%		33	19.60%	
2	812	20.70%	61	17.60%		29	17.30%	
3	1330	33.90%	117	33.80%		59	35.10%	
4	989	25.20%	114	32.90%		47	28.00%	
N-stage					< 0.001			< 0.001
0	391	10.00%	10	2.90%		9	5.40%	
1	1370	34.90%	57	16.50%		40	23.80%	
2	1543	39.40%	160	46.20%		66	39.30%	
3	616	15.70%	119	34.40%		53	31.50%	
Pre-NLR					0.001			0.17
<2.045	1980	50.90%	143	41.40%		75	45.50%	
≥2.045	1909	49.10%	202	58.60%		90	54.50%	
Pre-HB (g/L)					0.059			0.012
≤143	1920	49.40%	152	44.10%		65	39.40%	
>143	1970	50.60%	193	55.90%		100	60.60%	
Pre-PLR					0.067			0.220
<125	1981	50.90%	158	45.80%		76	46.10%	
≥125	1908	49.10%	187	54.20%		89	53.90%	
Pre-EBV DNA (copy/ml)					0.002			0.873
0	1011	26.70%	62	19.00%		42	26.10%	
>0	2782	73.30%	265	81.00%		119	73.90%	
Post-HB (g/L)					0.147			0.213
<122	1838	47.10%	177	51.20%		70	42.20%	
≥122	2065	52.90%	169	48.80%		96	57.80%	
Post-EBV DNA (copy/ml)					< 0.001			0.181
0	3208	89.80%	226	73.90%		127	86.40%	
>0	365	10.20%	80	26.10%		20	13.60%	

*Abbreviations*: *Pre-NLR* neutrophil to lymphocyte ratio before treatment, *Pre-HB* hemoglobin before treatment, *Pre-PLR* platelet to lymphocyte ratio before treatment, *Pre-EBV DNA* Epstein-Barr virus DNA before treatment, *Post-HB* hemoglobin after chemoradiotherapy, *Post-EBV DNA* Epstein-Barr virus DNA after chemoradiotherapy, *NMM* non-metachronous metastasis, *EMM* early metachronous metastasis (metastasis within 2 years after treatment), *LMM* late metachronous metastasis (metastasis beyond 2 years after treatment)

**Fig. 2:**
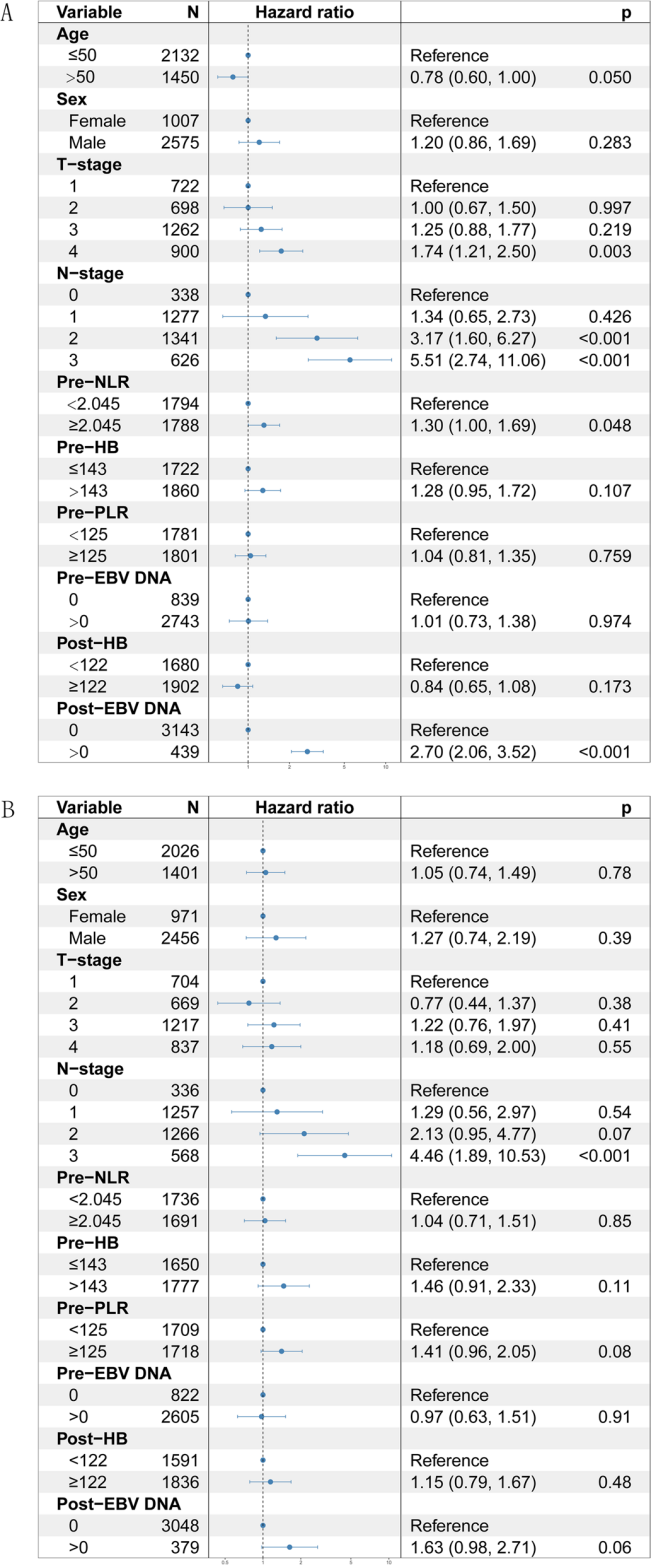
Multivariate Cox analysis of all risk factors. **A** Multivariate Cox analysis for the early metachronous metastasis (EMM) group. **B** Multivariate Cox analysis for the late metachronous metastasis (LMM) group

Multivariate Cox analysis showed that age (*p* = 0.05), T-stage (*p* = 0.003), N-stage (*p* < 0.001), pre-NLR (*p* = 0.048), and post-EBV DNA level (*p* < 0.001) were independent prognostic factors in the EMM group (Fig. 2A). Meanwhile, it revealed that N-stage (*p*<0.001), pre-PLR (*p* = 0.08) and post-EBV DNA (*p* = 0.06) were independent prognostic factors in the LMM group (Fig. 2B). In addition, Chi-squared test of the number of chemotherapy drugs and timing of chemotherapy between EMM group and LMM group showed that chemotherapy is not a prognostic factor for early or late metachronous metastasis in our research (Supplementary Tables 1 and 2).

### Calculate the attribution risk to metastasis

**Fig. 3:**
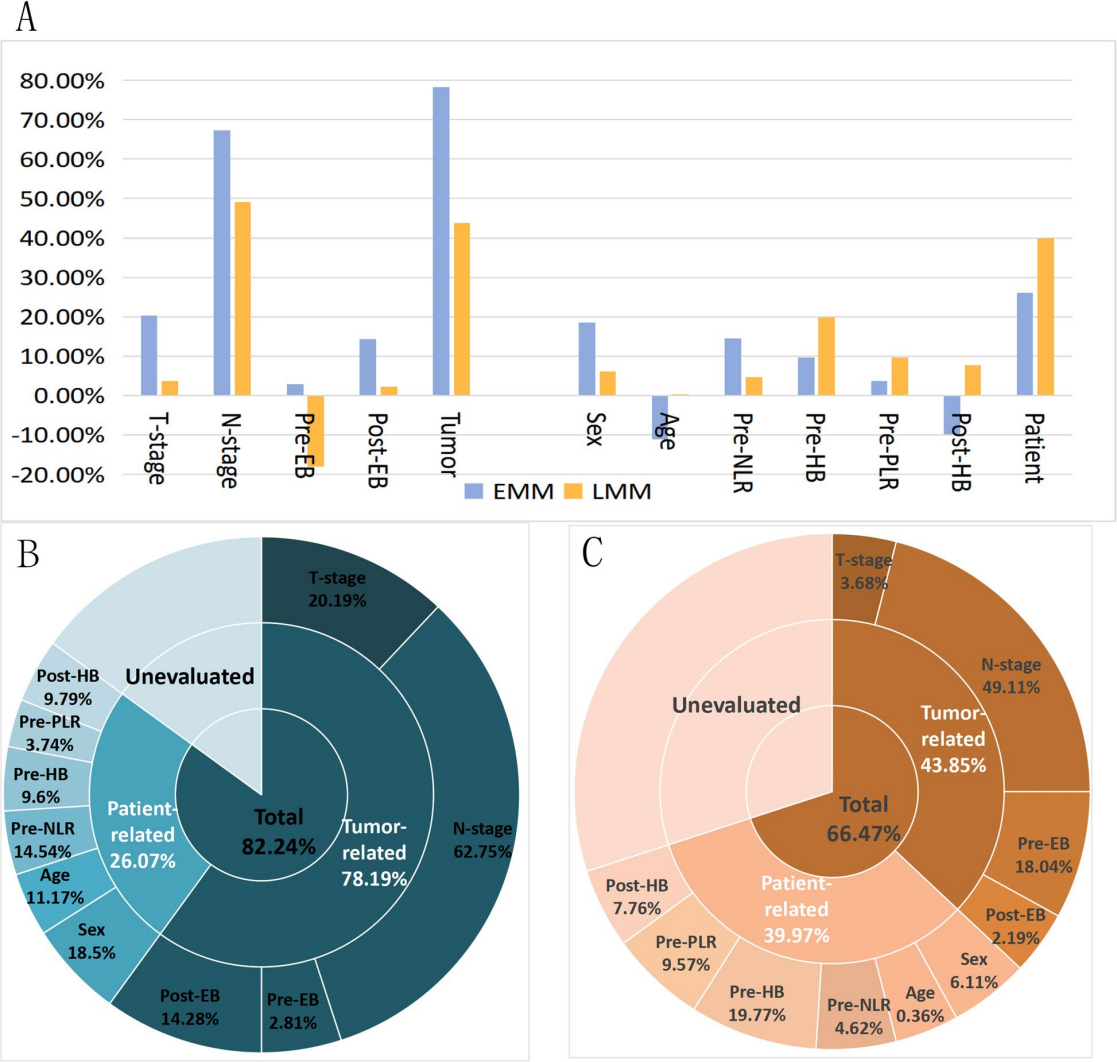
**A** The attributable risks for every individual risk factor and the combinations of tumor- and patient-related factors in the EMM and LMM groups. **B** The attributable risks in the EMM group. **C** The attributable risks in the LMM group

In epidemiology, PAR is a weight index for the statistical analysis of population exposure to some risk factors, which are attributed to these risk factors in the event of disease or death. Several risk factors have been identified for metastatic NPC in our study, and the IRAP was used to comprehensively examine the population attributable risks for every individual risk factor for metastasis in different periods (Table 3). The attributable risks of T-stage, N-stage, pre-EBV DNA, post-EBV DNA, age, sex, pre-NLR, pre-PLR, pre-HB and post-HB for early metastasis were 20.19, 67.25, 2.81, 14.28, 18.50, - 11.17%, 14.54, 9.60, 3.74% and - 9.79%, respectively, in the EMM group. The attributable risks of T stage, N stage, pre-EBV DNA, post-EBV DNA, age, sex, pre-NLR, pre-PLR, pre-HB, and post-HB for late metastasis were 3.68, 49.11, - 18.04%, 2.19, 6.11, 0.36, 4.62, 19.77, 9.57, and 7.76%, respectively, in the LMM group (Fig. 3A).

**Table 3:** The attributable risks for every individual risk factors and the combinations of tumor-related factors and patient-related factors in EMM and LMM group

Categories	Factors	AR in EMM group		AR in LMM group	
Tumor	T-stage	78.19%	20.19%	43.85%	3.68%
	N-stage		67.25%		49.11%
	Pre-EB		2.81%		-18.04%
	Post-EB		14.28%		2.19%
Patient	Sex	26.07%	18.50%	39.97%	6.11%
	Age		-11.17%		0.36%
	Pre-NLR		14.54%		4.62%
	Pre-HB		9.60%		19.77%
	Pre-PLR		3.74%		9.57%
	Post-HB		-9.79%		7.76%

*Abbreviations*: *AR* attributable risks, *EMM* early metachronous metastasis (metastasis within 2 years after treatment), *LMM* late metachronous metastasis (metastasis beyond 2 years after treatment), *Pre-NLR* neutrophil-to-lymphocyte ratio before treatment, *Pre-HB* hemoglobin before treatment, *Pre-PLR* platelet-to-lymphocyte ratio before treatment, *Pre-EBV DNA* Epstein-Barr virus DNA before treatment, *Post-HB* hemoglobin after chemoradiotherapy, *Post-EBV DNA* Epstein-Barr virus DNA after chemoradiotherapy

We then estimated the attributable risks for the combinations of factors (Table 3). After classification and multivariable adjustment, Fig. 3B shows that the attributable risks of tumor-related factors accounting for early metastasis were 78.19%, and patient-related factors were 26.07% in the EMM group. Figure 3C shows that in the LMM group, the attributable risk of tumor-related factors accounting for late metastasis was 43.85%, while the risk of patient-related factors accounting for metastasis was 39.97%. Compared to the EMM group, the weight of tumor-related factors for metastasis decreased by 34.34% (from 78.19 to 43.85%), while the weight of patient-related factors increased by 13.9% (from 26.07 to 39.97%) in the LMM group. The total attributable risk of all tumor- and patient-related factors was 82.24% in the EMM group, while the weight was 66.47% (decreased by 15.77%) in the LMM group. Except for these identified tumor- and patient-related factors, other unevaluated factors played a more important role in patients diagnosed with late metastasis, with the weight increasing by 15.77%, from 17.76% in the EMM group to 33.53% in the LMM group.

## Discussion

At present, the 5-year failure rate for NPC is 15 to 30% [5, 12, 13],and the 5-year locoregional control rate is approximately 90% [14]. Approximately 70% of treatment failures are ascribed to distant metastasis [15]. The time to metachronous metastasis differed between the patients. A study showed that the NPC failure hazard rate did not decline in a linear manner but showed a sharp peak at 2 years, with up to 59% of events being occurred within the first 2 years after treatment [16]. Other studies have shown that the majority of metachronous metastases (73.5%) occur within the first 2 years after treatment [4]. Our study showed that 67.32% of metachronous metastasis cases occurred within the first 2 years, which is in line with previous research. The probability of nasopharyngeal carcinoma metastasis is different at different periods. It can be speculated that the mechanism of metastasis is different at different periods, and the corresponding risk factors and their weights may also differ. Based on previous studies and our investigation, we classified all cases of metastasis as EMM or LMM according to whether metastasis occurred within or beyond the 2-year period.

Several risk factors have been identified for the metastasis of NPC, including T stage, N stage, EBV-DNA, inflammatory indicators, nutritional index, and other clinical risk factors [17]. The above-mentioned risk factors, T and N stage, are the most frequently used in evaluating prognosis and guiding therapy and are also closely related to metastasis. In this study, we found that the corresponding proportion of T and N stage impacts on metastasis was the highest. The PARs of the T and N stages in the EMM group were 20.19 and 67.25% respectively. Although the proportion in the LMM group decreased, it remained at 3.68 and 49.11%, respectively. The attributable risks in the N stage were significantly higher than those of other risk factors. It can be presumed that a high N stage represents a strong ability of invasion and migration and is prone to distant metastasis.

Patients with the same stage T and N disease and the same treatment regimen had different times of distant metastasis, which indicates that there are other factors that promote the occurrence of distant metastasis. Previous studies have reported that plasma EBV DNA is significantly correlated with distant metastasis [18, 19]. In this study, compared with the EMM group, the weight of pre-EBV DNA decreased by 20.85% (from 2.81% to - 18.04%) in the LMM group, and that of post-EBV DNA decreased by 12.09% (from 14.28 to 2.19%), indicating that EBV DNA is an important parameter for predicting early metastasis, but has little significance in late metastasis. The attributable risk for post-EBV DNA was 14.28%, which was significantly higher than that for pre-EBV DNA (2.81%). Therefore, post-EBV DNA is a better predictor of early metastasis than pre-EBV DNA is. This may be explained by the fact that post-EBV reflects residual tumors, which suggests an extremely high risk of treatment failure and early metastasis [20].

Tumor-related factors, such as EBV DNA and T and N stages, contributed the most to NPC metastasis. Many studies have already shown that patient-related factors, including age, sex, pre-NLR, pre-HB, pre-PLR, and post-HB, are closely associated with metastasis in patients with NPC. However, these continuous variables are controversial and the accepted cut-off points are still not consistent. In addition, the values of continuous variables have a wide distribution, which easily leads to bias, so that the medians were chosen to balance the sample size between groups and transform them into categorical variables. The median values were as follows: age, 50 years; pre-NLR, 2.045; pre-HB, 143 g/L; pre-PLR, 125; and, pre-HB, 122 g/L. In this study, compared with the EMM group, the weight of sex decreased by 12.39% (from 18.5 to 6.11%) in the LMM group, and the weight of age increased by 11.53% (from - 11.17 to 0.36%) in the LMM group, which indicated that male and young patients were more prone to early distant metastasis. The inflammation-based index has also a significant effect on metastasis in NPC. Compared to the EMM group, the attributable risk of pre-NLR decreased by 9.92% (from 14.54 to 4.62%) in the LMM group, and the proportion of pre-PLR increased by 5.83% (from 3.74 to 9.57%) in the LMM group. Immune and inflammatory responses in the microenvironment play critical roles in metastasis [21]. Neutrophils, a type of inflammatory cells, are involved in different steps of tumor development through the production of a variety of cytokines, and the release of angiogenic factors. In contrast, lymphocytes are also responsible for immune surveillance to remove tumor cells. Lymphocytes, which are an essential component of host immunity, play a critical role in the destruction of residual tumor cells and related micrometastases [21–28]. Neutrophils, lymphocytes, and platelets may cause tumor invasion and metastasis by affecting the tumor microenvironment and immune system. HB, a nutritional index, also affects the prognosis [29]. Compared with the EMM group, the weight of Pre-HB increased by 10.17%, from 9.6 to 19.77%, while the proportion of Post-HB increased by 17.55%, from - 9.79 to 7.76%, in the LMM group. Patients with poor nutritional status are more likely to develop early metastasis, whereas metastasis appears later in patients with good nutrition.

After classification and multivariable adjustment, compared with the EMM group, the total attributable risks for the combinations of tumor-related factors decreased by 34.34% (from 78.19 to 43.85%) in the LMM group, whereas the total AR of patient-related factors increased by 13.9% (from 26.07 to 39.97%) in the LMM group. Tumor-related factors contributed the most to the prediction of early metastasis in patients with NPC. However, in patients with late-metastasis NPC, the weight of tumor- and patient-related factors decreased and increased significantly, respectively. In addition, except for these identified tumor- and patient-related factors, other unevaluated factors played a more important role in patients with late metastasis, with the weight increasing by 15.77%, from 17.76% in the EMM group to 33.53% in the LMM group. Combined with the above results, we can assume that the related factors affecting metachronous metastasis in NPC are different; at least the attributable risks and influence are different in different periods. The risk factors included in this study for NPC metastasis have been confirmed by previous studies, which can explain the metastatic risk in most early metastatic cases but are insufficient for cases of late metastasis. Therefore, further investigation of other risk factors associated with LMM is necessary.

The primary approach to cure NPC is local radiotherapy. Tumorigenesis involves multiple genes and is a complex biological process that involves multiple stages and steps. Circulating tumor cells are present in the blood circulation of patients with NPC [30, 31]. After systematic treatment, the vast majority of tumors are killed and very few tolerant cells remain dormant [32, 33]. In patients with early metastasis, we can consider that the remaining dormant tumor cells do not enter a dormant state, which means that cell division and apoptosis are still imbalanced and only delay tumor cell division and growth rate. Metastasis eventually occurred over time. Tumor cell tolerance and the tumor microenvironment play a leading role in this process. In patients with late metachronous metastasis, we consider that the remaining tumor cells are possibly long dormant or homeostatic; when external factors affect the internal environment, the homeostasis will be broken and the dormant tumor cells will grow and metastasize. In other words, low-intensity maintenance therapy may be necessary for patients who are prone to early metastasis, whereas general preventive measures, such as a healthy lifestyle and good nutrition, may be effective for those with late metastasis.

This study has two limitations that need to be addressed. First, the retrospective studies may have been biased. However, the large sample size may reduce the bias from retrospective data. Second, this study explored the difference and the weight of risk factors of metastasis in different periods but did not establish a model to predict the risk for metastasis in patients.

## Conclusion

Most distant metastases of NPC occur within the first 2 years after treatment. Early metastasis is mainly affected by tumor-related factors, which account for a declining percentage of LMM cases. Previously identified risk factors can explain the metastatic risk in most early metastasis cases; however, they are insufficient for cases of late metastasis. Further investigation is necessary to explore the other risk factors associated with LMM.

## Supplementary Information

**Additional file 1: Supplementary Figure 1.** The flow chart of the inclusion for patients.

**Additional file 2: Supplementary Table 1.** Chi-squared test of the number of chemotherapy drugs between early metachronous metastasis (EMM) group and late metachronous metastasis (LMM) group.

**Additional file 3: Supplementary Table 2.** Chi-squared test of the timing of chemotherapy drugs between EMM group and LMM group.

## Data Availability

Data are available upon reasonable request. The data sets generated during and/or analyzed during the current study are available from the corresponding author on reasonable request.

## Abbreviations

NPC: Nasopharyngeal carcinoma
IMRT: Intensity-modulated radiotherapy
EBV: Epstein-Barr virus
HB: Haemoglobin
NLR: Neutrophil to lymphocyte ratio
PLR: Platelet to lymphocyte ratio
PARs: Population attributable risks
AJCC: American Joint Committee on Cancer
IRAP: Interactive Risk Attributable Program
AR: Attribution risk
EMM: Early metachronous metastasis
LMM: Late metachronous metastasis
NMM: Non-metachronous metastasis
CTCs: Circulating tumor cells
