# Supplementary figures and images for: Analysis of risk characteristics for metachronous metastasis in different period of nasopharyngeal carcinoma
Source: BMC Cancer. 2023 Feb 17;23:165. doi: 10.1186/s12885-023-10641-8 (PMC9938628; doi:10.1186/s12885-023-10641-8)

**Supplementary Figure 1.** The flow chart of the inclusion for patients.


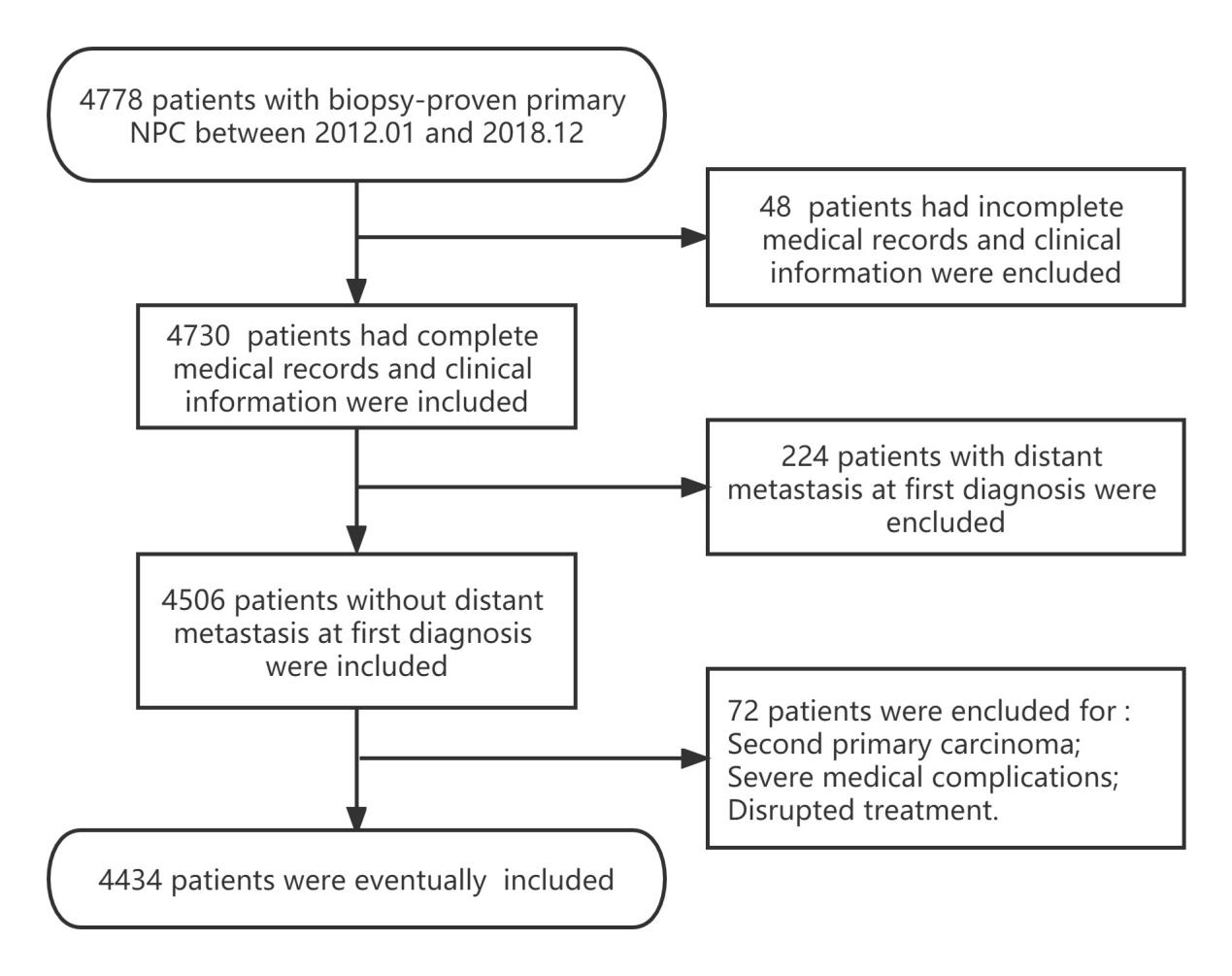

Supplement: Supplementary file 1 — Additional file 1: Supplementary Figure 1. The flow chart of the inclusion for patients. [file 12885_2023_10641_MOESM1_ESM.docx]
