# Supplementary material for: Analysis of risk characteristics for metachronous metastasis in different period of nasopharyngeal carcinoma
Source: BMC Cancer. 2023 Feb 17;23:165. doi: 10.1186/s12885-023-10641-8 (PMC9938628; doi:10.1186/s12885-023-10641-8)
Supplement: Supplementary file 2 — Additional file 2: Supplementary Table 1. Chi-squared test of the number of chemotherapy drugs between early metachronous metastasis (EMM) group and late metachronous metastasis (LMM) group. [file 12885_2023_10641_MOESM2_ESM.docx]

**Supplementary Table 1.** Chi-squared test of the number of chemotherapy drugs between early metachronous metastasis (EMM) group and late metachronous metastasis (LMM) group.

| Number of drugs | 1 drug | 2 drugs | P value |
| --- | --- | --- | --- |
| EMM group  (n=346) | 12 | 334 | 0.113 |
| LMM group  (n=168) | 11 | 157 |  |
| TOTAL | 23 | 491 |  |

CCRT alone is 1 drug

IndCT+CCRT is 2 drugs

IndCT+CCRT+adjCT is 2 drugs
