# Supplementary material for: Analysis of risk characteristics for metachronous metastasis in different period of nasopharyngeal carcinoma
Source: BMC Cancer. 2023 Feb 17;23:165. doi: 10.1186/s12885-023-10641-8 (PMC9938628; doi:10.1186/s12885-023-10641-8)
Supplement: Supplementary file 3 — Additional file 3: Supplementary Table 2. Chi-squared test of the timing of chemotherapy drugs between EMM group and LMM group. [file 12885_2023_10641_MOESM3_ESM.docx]

**Supplementary Table 2.** Chi-squared test of the timing of chemotherapy drugs between EMM group and LMM group.

| Treatment | IndCT+CCRT | IndCT+CCRT+adjCT | P value |
| --- | --- | --- | --- |
| EMM group  (n=346) | 291 | 43 | 0.878 |
| LMM group  (n=168) | 136 | 21 |  |
| TOTAL | 427 | 64 |  |
